# Designing coarse grained-and atom based-potentials for protein-protein docking

**DOI:** 10.1186/1472-6807-10-40

**Published:** 2010-11-15

**Authors:** Dror Tobi

**Affiliations:** 1 Department of Computer Sciences and Mathematics, Department of Molecular Biology, Ariel University Center of Samaria, Ariel 40700, Israel

## Abstract

**Background:**

Protein-protein docking is a challenging computational problem in functional genomics, particularly when one or both proteins undergo conformational change(s) upon binding. The major challenge is to define a scoring function soft enough to tolerate these changes and specific enough to distinguish between near-native and "misdocked" conformations.

**Results:**

Using a linear programming (LP) technique, we developed two types of potentials: (i) Side chain-based and (ii) Heavy atom-based. To achieve this we considered a set of 161 transient complexes and generated a large set of putative docked structures (decoys), based on a shape complementarity criterion, for each complex. The demand on the potentials was to yield, for the native (correctly docked) structure, a potential energy lower than those of any of the non-native (misdocked) structures. We show that the heavy atom-based potentials were able to comply with this requirement but not the side chain-based one. Thus, despite the smaller number of parameters, the capability of heavy atom-based potentials to discriminate between native and "misdocked" conformations is improved relative to those of the side chain-based potentials. The performance of the atom-based potentials was evaluated by a jackknife test on a set of 50 complexes taken from the Zdock2.3 decoys set.

**Conclusions:**

Our results show that, using the LP approach, we were able to train our potentials using a dataset of transient complexes only the newly developed potentials outperform three other known potentials in this test.

## Background

Proteins interacting with other proteins are known to play key roles in almost all cellular and biological processes such as signalling, metabolism, and trafficking. Therefore, understanding protein-protein interactions is fundamental to our ability to comprehend and control cell function. Protein complexes can be either permanent or transient. Polypeptide chains of permanent (obligatory) complexes remain bound to each other throughout their functional lifetime. An example hereof is the complex between β and γ subunits of hetero-trimeric G-proteins [1]. In transient complexes, the subunits can either form a complex or detach from each other as a result of specific biological conditions. The overall structures of these proteins are stable in the unbound as well as in the bound state. The complex of cyclin and cyclin-dependent protein kinase is an example of such a transient complex [2].

The molecular mechanism of interactions between protein-protein complexes is usually characterized by structure determination methods such as X-ray crystallography, NMR spectroscopy, and cryo-electron microscopy. However, many complexes are too transient to lend themselves to experimental characterization. Docking simulations have recently gained importance as possible means for predicting the quaternary structures of protein-protein complexes [3]. Yet, these algorithms generate a large number of false-positives and false-negatives, mainly due to the inaccuracy of scoring functions (docking potentials) used to evaluate the docked conformations [4-6].

Docking algorithms that reconstruct known complexes using the structures of the proteins in the bound form (*bound docking problem*) generally show reasonable levels of success. However, when the structures in the unbound form are used as input (*unbound docking problem*), the performance of the same algorithms becomes rather poor. This failure is mainly attributed to the inability of the algorithms and the scoring functions to take account of the conformational changes that (may) accompany complex formation [4]. In an effort to overcome these deficiencies, one usually resorts to additional biological data, which assist in singling out the native (like) conformation(s) among putative docked conformations [7]. However, in the absence of prior knowledge regarding the binding site, exhaustive searches of up to 10^9 ^different positions and orientations are performed for the substrate [8], followed by a refinement and clustering stage. Thus, it is difficult to take into account all the possible changes in internal conformations that can facilitate binding [5], and detect the optimal bound state. Currently, protein-protein docking methods are often successful if the experimentally determined partner proteins undergo little conformational changes upon binding. However, the realistic and computationally efficient treatment of conformational changes, especially of the protein backbone, during docking remains a challenge. New promising approaches of flexible refinement, ensemble docking and explicit inclusion of flexibility during the entire docking process, have been developed [9].

Various strategies have been adopted to score putative protein-protein docked complexes. Most of them utilize atomic level potentials [10-16], other employ residue level potentials [17-20], or a combination of both [8,21,22]. Many of these potentials are knowledge-based [8,13,15,17-21,23,24], others are force field-based [12,25,26], or a combination of force field and knowledge-based potentials [10,14,22].

The knowledge-based approach aims at deriving empirical interaction parameters from known structures. The accuracy of these potentials depends on the ability to obtain reliable statistics and to correctly define the reference state. The relatively small number of solved transient protein-protein complexes, and the small number of contacts at the interface of these complexes result in poor statistics [27]. Therefore, many knowledge-based potentials were derived from a dataset of protein folds (intra-protein contacts) [8,13,21] or from datasets of transient and non-transient interfaces [17,19,20,22,23].

Several works show differences in amino acid composition between protein folds, transient interfaces and non-transient interfaces [27-30]. Ofran and Rost [29] performed a comprehensive survey of proteins, identifying six types of interfaces, each one significantly different in its amino acid composition and pairing preference. These differences impair the ability to obtain reliable statistics and to define accurate reference states, which may well be type specific. Consequently, potentials solely derived from a dataset of *transient *protein complexes may be more suitable for docking simulations as only the transient complexes may need computational docking predictions. Further more, the LP technique does not require the definition of a reference state therefore the resulting potentials may not be biases toward a specific type of transient complex as shown by Liu *et al *[31].

Our purpose is to develop knowledge-based protein-protein docking potentials (DPs) tailored to transient complexes that will efficiently discriminate between the structures of native and non-native complexes. Therefore, the potentials are exclusively derived from a dataset of transient complexes. For each complex, a large number of misdocked (decoy) complexes are generated. A set of potentials is optimized using an LP technique such that for each complex the native state has a lower energy than any of its decoys. This approach enables us to include in our training set large amount of false positives (FPs) in addition to the true positives (TPs), thus alleviating the statistical problem of limited data. In the present study we develop two sets of potentials, coarse-grained and atom-based, using a functional form of the potentials that is not highly sensitive to small structural changes.

This work is an extension of our previous work [18] on designing coarse-grained protein-docking potentials. However, in our current study we developed two-step potentials, because we are using a larger dataset that cannot be solved using single-step potentials. The applicability of the new set of DPs is illustrated and compared by performing a jackknife test on the Zdock 2.3 decoys set.

### Outline of the algorithm

#### The linear programming approach

We proposed to overcome the sampling problem for deriving DPs by applying an LP technique to a set of transient complexes. The utility of this technique in developing both folding and docking potentials previously has been exemplified, by us [18,32] and by others [33], after the pioneering work of Maiorov and Crippen [34]. For each complex we generated a large number of putative docked conformations, a few compliant with the native (or native-like) conformation(s), and the remaining '*misdocked'*. The basic requirement from the DP is to yield, for each native complex, a free energy lower than that of *any *misdocked complex. Each of the non-native decoys defines a constraint (in the form of an inequality) on the energy function and we are looking for a set of potentials satisfying these constraints. The advantages of this approach over the statistical approaches are twofold: (a) Statistical approaches learn from known native states on other native states, i.e. the (inverse) Boltzmann statistics is applied to a set of known protein complexes in order to infer the structure of other native complexes. In the LP approach, we learn from a set of native states and large sets of non-native states (FPs) on how a native state should, and should not, look like. Therefore, we have more varied data to derive the empirical potentials. (b) The LP approach is *not *sensitive to over-or under-representation or sequence/structure homologies in the training set.

#### Protein models

The *side chain based model *was previously used by us to develop docking potentials [18]. In short, each amino acid is represented by *three *interaction sites: the side chain centroid (S), the amide nitrogen (N) and the carbonyl oxygen (O) on the backbone (B). This representation involves six types of interactions, S-S, S-O, S-N, O-N, N-N and O-O, the first three being residue-specific. This results in a set of 210 (S-S) + 20 (S-0 + S-N) + 1 (O-N + N-N + O-O) = 253 types of interactions. The *atom based model *was first introduced by Zhang *et al*. [13]. Each side chain is represented by all its heavy atoms which are divided into eighteen types (see Table 1). This model results in 171 different interaction types.

**Table 1:** The designation of the 18 atom types ^1^

Atom type	Amino acid	Atom^3^	Atom type	Amino acid	Atom	Atom type	Amino acid	Atom
N	BKBN^2^	N		Glu	CD		Leu	CG
Cα	BKBN	CA		Glu	OE1		Lys	CG
C	BKBN	C		Glu	OE2		Met	CG
O	BKBN	O	RNη	Arg	CZ		Met	SD
GCα	Gly	CA		Arg	NH1		Phe	CG
Cβ	Ala	CB		Arg	NH2		Phe	CD1
	Arg	CB	NNδ	Asn	CG		Phe	CD2
	Asn	CB		Asn	OD1		Phe	CE1
	Asp	CB		Asn	ND2		Phe	CE2
	Cys	CB		Gln	CD		Phe	CZ
	Gln	CB		Gln	OE1		Thr	CG2
	Glu	CB		Gln	NE2		Trp	CG
	His	CB	RNε	Arg	CD		Trp	CD1
	Ile	CB		Arg	NE		Trp	CD2
	Leu	CB	SOγ	Ser	CB		Trp	CE2
	Lys	CB		Ser	OG		Trp	CE3
	Met	CB		Thr	OG1		Trp	CZ2
	Phe	CB		Tyr	OH		Trp	CZ3
	Pro	CB	HNε	His	CG		Trp	CH2
	Pro	CG		His	ND1		Tyr	CG
	Pro	CD		His	CD2		Tyr	CD1
	Thr	CB		His	CE1		Tyr	CD2
	Trp	CB		His	NE2	LCδ	Ile	CG2
	Tyr	CB		Trp	NE1		Ile	CD
	Val	CB	YCξ	Tyr	CE1		Leu	CD1
KNξ	Lys	CE		Tyr	CE2		Leu	CD2
	Lys	NZ		Tyr	CZ		Met	CE
KCδ	Lys	CD	FCξ	Arg	CG		Val	CG1
DOδ	Asp	CG		Gln	CG		Val	CG2
	Asp	OD1		Glu	CG	CSγ	Cys	SG
	Asp	OD2		Ile	CG1			

1. Atom types were taken from the work of Zhang *et al*. [13].

2. BKBN designate main chain backbone atoms.

3. Atom names are according to the PDB nomenclature.

#### Energy function and parameters optimization

The DP design problem is set as follows: Assume *X *to be the coordinate vector representing a protein structure, (${\hat{X}}_{a}:{\hat{X}}_{b}$) the coordinates of the native complex (composed of monomers *a *and *b*), and ${\left\{{\hat{X}}_{a}:X_{b}^{i}\right\}}_{i=1}^{N}$ the set of *N *decoys generated by alternative rotations and translations of monomer *b*. The demand upon the energy of the native complex is to be lower than that of any of the misdocked conformations, as expressed by the set of N inequalities

(1) $${\left\{U\left({\hat{X}}_{a}:X_{b}^{i}\right)\text{-}U\left({\hat{X}}_{a}:{\hat{X}}_{b}\right)\;>\;\varepsilon_{i}\right\}}_{i=1}^{N}$$

where *U(X_a_:X_b_) *represents the potential energy of a complex and ε*_i _*is a nonnegative constant, set to 0.1 in the calculations below.

#### Parameter optimization

The total energy is expressed as a linear combination of a parameter set ${\left\{p_{l}\right\}}_{l=1}^{L}$

(2) $$U\left(X_{a}:X_{b},P\right)=\sum_{l=1}^{L}S_{l}\left(X_{a}:X_{b}\right)p_{l}$$

where ${\left\{S\left(X_{a}:X_{b}\right)\right\}}_{l=1}^{L}$ designates the set of L (L = 253 and 171 for the side chain-based and atom-based model basis functions, respectively). The functions ${\left\{p_{l}\right\}}_{l=1}^{L}$ are linear and, therefore, can be optimized using LP algorithms such as Simplex or Interior-Point. The functions $S_{l}\left({\hat{X}}_{a}:{\hat{X}}_{b}\right)$ are expressed in terms of a position (*r_n_*) dependent step function

(3) $$H^{l}(|r_{n}-r_{m}|)=\{\begin{array}{c} 1\text{if}|r_{n}-r_{m}|\leq r_{cutoff}^{l}\\ 0\;otherwise \end{array}$$

that extracts all interacting pairs *m *and *n *(in the respective monomers *a *and *b*) within an interaction range *r^l^_cutoff _*characteristic of the specific type (*l*) of interaction, i.e.

(4) $$S_{l}(X_{a}:X_{b})=\sum_{n\in a,m\in b}H^{l}\left(\left|r_{n}-r_{m}\right|\right)\delta (l,nm)$$

Hereδ(*l,nm*) is the Kronecker delta, which equals 1 if the type of interaction between sites *m *and *n *is of type *l*, and 0 otherwise. For the side chain-based model we adopted $r_{cutoff}^{l}$ values of 4.4 Å for B-B, 5.6 Å for B-S, and 6.8 Å for S-S interactions, whereas for the atom-based model we adopted an $r_{cutoff}^{l}$ value of 6.0 Å. The total energy for a given decoy *i *is reduced to the summation over the effective *P_l_*, weighted by the numbers $n_{l}^{i}$ of contacts of type *l *occurring in the examined decoy. The *N *inequalities in Eq 1 are thus replaced by

(5) $${\left\{\sum_{l=1}^{253}p_{l}(n_{l}^{i}-{\hat{n}}_{l})>\varepsilon_{i}\right\}}_{i=1}^{N}$$

where ${\hat{n}}_{l}$ is the number of contacts of type *l *in the native complex.

With one step contact potentials, a single cutoff distance defines the threshold below which interactions are considered to be "on", and above which these are considered to be "off". Such potentials may be too simplified to accurately describe the binding energy of interaction proteins. Alternatively, one can design multi-step contact potentials. We replaced the step function $H^{l}\left(\left|r_{n}-r_{m}\right|\right)$ by$H^{l,d}\left(\left|r_{n}-r_{m}\right|\right)$, where the superscript *d *refers to different distance ranges (*r*_1_-*r*_2_, *r*_2_-*r*_3_, ..., *r*_D-1_-*r*_D_). The energy of the *i*^th ^decoy is computed from the summation over the effective docking potentials *p_l,d_*, weighted by the numbers $n_{l,d}^{i}$ of contacts of type *l *occurring at the distance interval (*d,d+*1] in the examined decoy. The *N *inequalities (eq 5) are thus replaced by

(6) $${\left\{\sum_{l=1}^{253}\sum_{d=1}^{D}p_{l,d}(n_{l,d}^{i}-{\hat{n}}_{l,d}^{})>\varepsilon_{i}\right\}}_{i=1}^{N}$$

Determining the cutoff value for distance ranges in multiple step potentials is not trivial. To overcome this problem we derived a set of single step potentials using a series of successive cutoff values and measuring the correlation coefficients between potentials with adjacent cutoff values. If the correlation is relatively low another step is created at this point. In the side chain based-models the following steps were used: *r_1 _*≤ 4.5 and 4.5 < r_2 _≤6 Å for B-B, *r_1 _*≤ 5.5 and 5.5 <*r_2 _*≤7 Å for B-S, and *r_1 _*≤ 6.5 and 6.5 <*r_2 _*≤8.0 Å for S-S interactions to get a set of 253 × 2 = 506 parameters. In the atom-based models the following steps were used: *r_1 _*≤ 4 and 4<*r_2 _*≤6 Å to get a set of 171 × 2 = 342 parameters.

#### Generating a population of putative docked conformations

Decoys for the complexes, in their bound state, were generated in the dataset *Protein-Protein Docking Benchmark 2.0 *by Mintseris *et al*. [35] (dataset 1). This dataset includes 83 Protein Data Bank (PDB) entries. We adopted the docking algorithm of Palma *et al*. [8], taking into consideration only the surface matching criterion to generate docked conformations. Geometric fit was assessed from the number of overlapping *surface nodes *between the receptor (large protein) and the substrate (small protein). The use of this criterion alone in the docking process enables us to generate an unbiased set of decoys, which form many contacts between the receptor and the ligand yet lacking any physicochemical basis. For any given rotation angle, all possible docking positions were divided into cells of 3 × 3 × 3 nodes according to their translation vector, and the best matching position from each cell was preserved. This modification of the algorithm enabled us to generate a comprehensive and unbiased set of misdocked conformations for each complex. To reduce the large numbers of decoys generated for each complex, the conformations were further clustered using a hierarchical clustering algorithm applying a cutoff value of root mean square deviation (RMSD) ≤ 10 Å and the central conformation of each cluster was preserved. On average, 144,000 non-native decoys (RMSD > 5.0 Å) were generated for each complex, which summed up to a total of 11,973,763 non-redundant inequalities or constraints on the potentials' parameters. Another set of complexes (dataset 2), listed in Table 2, is based on the work of Lo Conte *et al*. [27]. This set contains 40 complexes for which previously [18] we had generated decoys excluding those that share more that 60% sequence identity (receptor and ligand) with any of the complexes in dataset 1. Dataset 3 was derived from the dataset of Mintseris and Weng [36] as explained in the Results section (see Table 3) and decoys were generated as above.

**Table 2:** Dataset 2 contains 40 complexes ^1^

1A0O	1EFN	1KB5	1TOC
1AGR	1FIN	1MEL	1TX4
1BRS	1FLE	1MKW	1YCS
1BTH	1FSS	1NFD	1YDR
1CBW	1GLA	1NMB	2KAI
1CHO	1GUA	1OSP	2PTC
1CSE	1HWG	1PPF	2TRC
1DHK	1IAI	1STF	3SGB
1DVF	1IGC	1TBQ	4CPA
1EBP	1JHL	1TGS	4HTC

1. A subset of the Lo Conte *et al*. [27] dataset. The table list PDB codes.

**Table 3:** Dataset 3 contains 38 complexes ^1^

1TNR	1FJ1	1HEZ	1HX1
1IM3	1SLU	1WWW	1G4Y
1DF9	1CXZ	1IM9	1CIC
1BDJ	1DX5	1EO8	1JMA
1GOY	1C4Z	1ADQ	1I8L
1GH6	1FNS	1JDP	1D2Z
1E0O	1BZQ	1DEE	1AVG
3YGS	1F02	2VIR	1AVA
1NRN	1EBD	1QFU	
1QO3	1J7V	1IAR	

1. A subset of the dataset of 209 transient complexes compiled by Mintseris and Weng [36]

#### Numerical solution of linear inequalities

Inequalities were solved as explained in our previous work [18]. In short, each of the inequalities divides the parameter space into two regions, one allowed (any point within this space represents a valid solution), and one forbidden. A given inequality may affect the solution in three different ways: It may (1) further restrict the space allowed for the parameter set (most desirable), (2) have no effect on the allowed space, or (3) impose an impossible condition (reducing the allowed parameter space to zero). In the latter case no parameter set could satisfy all inequalities, making the inequalities unsolvable. Linear inequalities were solved using the interior point program BPMPD [37].

#### Test decoys

Test decoys were used to compare the performance of resulting potentials in the bound and unbound states. Decoy sets containing near-native complexes were generated for the complexes in dataset 1 (*Benchmark 2.0 *by Mintseris *et al*. [35]). This dataset contains the bound and unbound structures of each of the complexes' subunits. Near-native decoys are defined as complexes having an RMSD ≤ 5.0 Å from the native state. To each complex, docking simulations close to the native state were performed and near-native decoys were generated. Docking was performed using the algorithm of Palma *et al*. [8], taking into consideration only the surface matching criterion to generate docked conformations. Geometric fit was assessed from the number of overlapping *surface nodes *between the receptor and the substrate. The docking was performed close to the native state by rotating the substrate by up to 30° and translating by up to 5Å around the native state. These decoys were clustered using a cutoff RMSD value of ≤ 3 Å and the central conformation of each cluster was preserved. Subsequently, the preserved decoys were sorted according to their RMSD values from the native state, and a subset of on average 95 decoys, uniformly spanning the entire range of near-native decoys, was selected. This subset was combined with the above set of non-native decoys generated for each complex. Test decoys in the unbound state were generated by superimposing the unbound structures on the decoys generated in the bound state. By doing so we generated for each complex a set of decoys containing a representative set of both non-native and near-native decoys that enabled us to accurately compare the performance of the potentials in the *bound *and *unbound *states irrespective of the docking sampling problem.

#### Jackknife procedure

The jackknife test was performed each time by randomly selecting ten complexes from dataset 1. Thereupon, any complex having more than 35% sequence identity with the selected complexes was removed from datasets 1, 2, and 3, and the remaining complexes served as the training set. New potentials were optimized using the reduced training set and tested on the selected ten complexes.

## Results

### Optimizing docking potentials

**Figure 1:**
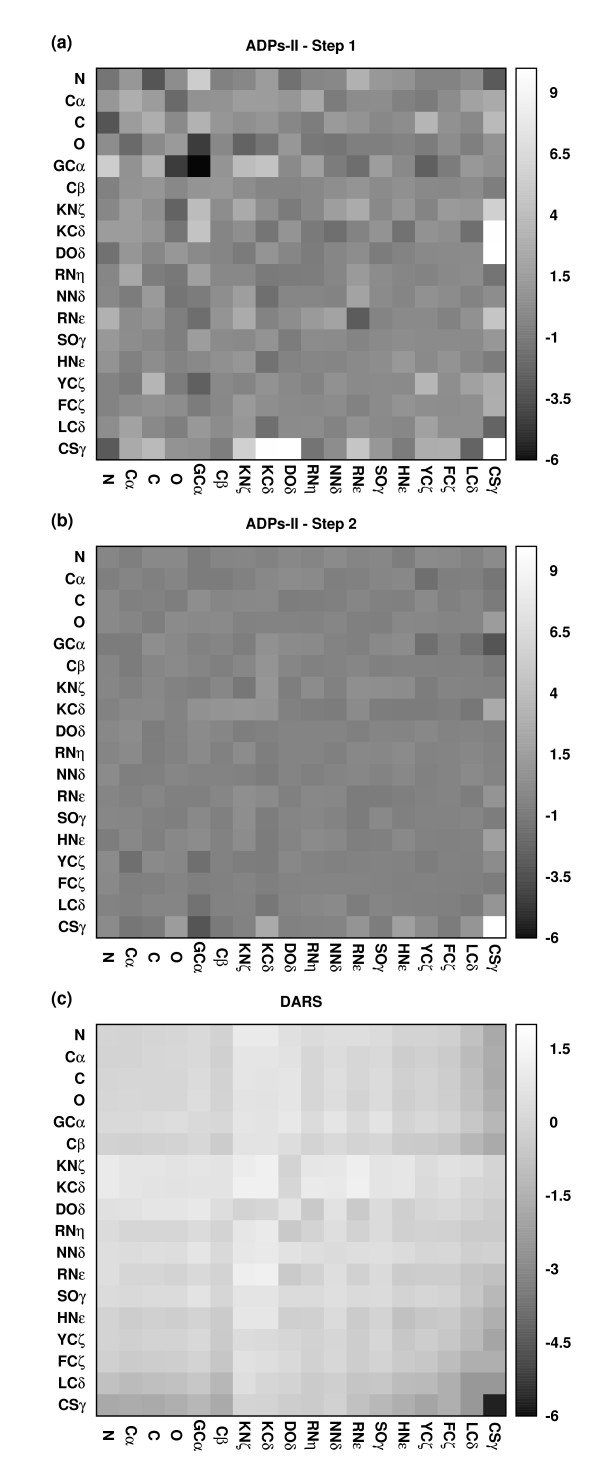
**Inter-atomic contact energies for protein-protein docking**. The maps corresponding to the first and second step of the presently obtained Atomic Docking Potentials-II are shown in Panels (a) and (b), respectively. Panel (c) shows the map of the DARS potentials [15].

**Figure 2:**
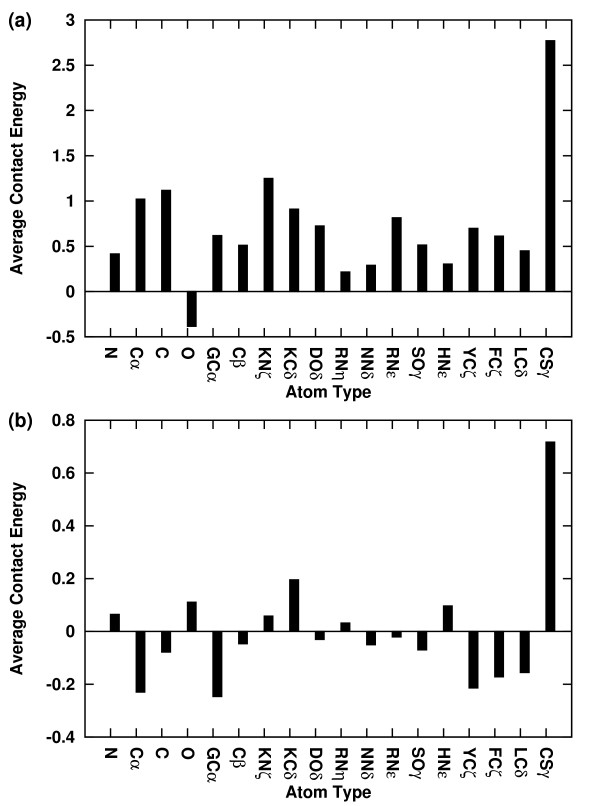
**Average inter-atomic contact energy per atom type for the first (Panel a) and second (Panel b) step**. Averaging was done over all atom types.

When optimizing the single-step potentials using constraints derived from dataset 1 we encountered a state of infeasibility both for the atom-based and the side chain-based models. That is, no set of parameters could satisfy all the constraints. Therefore, two-steps potentials were derived. With these potentials we were able to solve all the constraints that were derived from both dataset 1 and dataset 2. The resulting SDPs and initial set of ADPs (ADPs-I) are presented in the additional file 1 Tables S1 and S2, respectively. Next, we scanned the dataset of transient complexes compiled by Mintseris and Weng [36] and identified a subset of 38 complexes (dataset 3, see Table 3), for which not all constraints were satisfied using the present ADPs-I and added them to our training dataset. Thereupon, we solved all constraints derived from datasets: 1, 2, and 3 to obtain a new set of atomic contact potentials (ADPs-II). ). The potentials are presented in Table S3 (additional file 1). In Figure 1, Panels (a) and (b) display the maps of the first and second steps of the ADPs-II, respectively, whereas Panel (c) displays the single step Decoys As the Reference State (DARS) [15] potentials. The average contact energy per atom group is displayed in Figure 2. The ADPs-II properties' and the qualitative differences between the two potentials will be discussed below. When attempting to solve these constraints using the side chain-based model a state of infeasibility was encountered, which persisted even when using a different set of cutoff values: *r_1 _*≤ 4.5 and 4.5 < r_2 _≤6 Å for B-B, *r_1 _*≤ 5.5 and 5.5 <*r_2 _*≤7.5 Å for B-S, and *r_1 _*≤ 6.5 and 6.5 <*r_2 _*≤9.0 Å for S-S interactions.

### Optimal tolerance to structural changes

**Figure 3:**
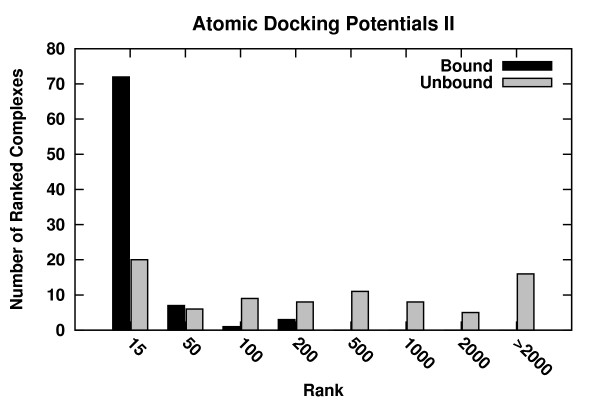
**Ranking success histogram of the ADPs-II using dataset 1 **[35]. For each complex decoys were ranked order according to the binding energy alone and the rank of the best (lowest energy) near-native (RMSD ≤5Å) decoy was counted. The histogram shows the number of complexes, whose hits were ranked at the top 15, 16-50, 51-100, 101-200, 201-500, 501-1000, 1001-2000, and > 2000 places in the bound (black) and unbound (gray) cases.

In order to test the optimal tolerance of the potentials to structural changes between the bound and unbound states of the proteins, the decoys (see Methods) were ranked order exclusively based on the binding-energies. This test enabled comparing the performances of the potentials in the *bound *and the *unbound *states regardless of the sampling. Ranking success for ADPs-II is shown in Figure 3. In the *bound *state a near-native decoy is ranked in the top fifteen places in 72 out of 83 cases, and in the top fifty in 79 out of 83 cases. In the top 200 places a near-native was identified in all cases. These results are expected since these complexes were included in the training dataset. When ranking the *unbound *decoys using ADPs-II, in the top fifteen a near-native complex was ranked in twenty cases, and in the top fifty in twenty six cases. In the top 2000 ranked decoys no near-native complex was detected in sixteen cases.

**Figure 4:**
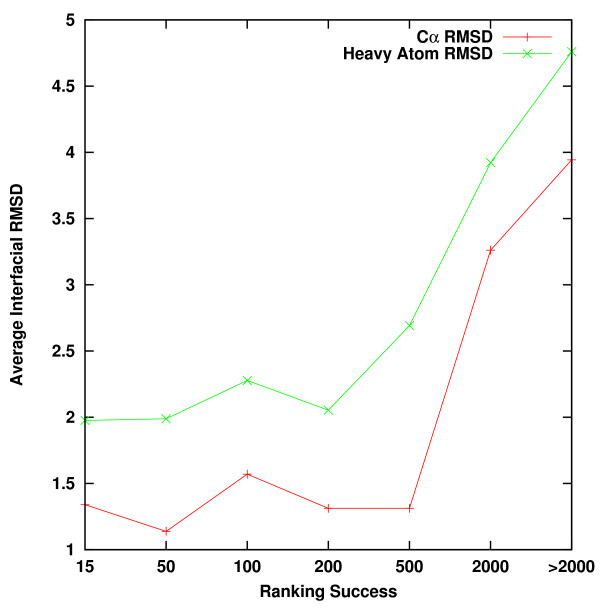
**Average interfacial RMSD of each ranked group plotted vs. the ranking success**. The average RMSD of interfacial amino acids was measured using C^α ^(red) and heavy-atoms (green). Interfacial amino acids are defined as those having at least one atom in close proximity (*r_c _*≤ 4.5 Å) to any atom of the other subunit.

In order to estimate the tolerance of the potentials to structural changes we measured the C^α ^RMSD and heavy-atom RMSD of the interfacial amino acids (IRMSD) between the bound and unbound structure. Interfacial amino acids are defined as those having at least one atom in close proximity (*r_c _*≤ 4.5 Å) to any atom of the other subunit. The average IRMSD vs. the ranking success of each ranked group is plotted in Figure 4. Complexes with a near-native structure ranked in the top 200 places do not show significant structural change between the bound and unbound states (average C^α^/heavy-atom IRMSD 1.35/2.05 Å). Complexes that are ranked in the range of 200-500 places are characterized by increased side chains displacements. Complexes with a near native structure not ranked in the top 500 places are characterized by large side chain and backbone movements (average C^α^/heavy-atom IRMSD > 1.95/2.69 Å).

### Rescoring ZDOCK2.3 decoys

**Figure 5:**
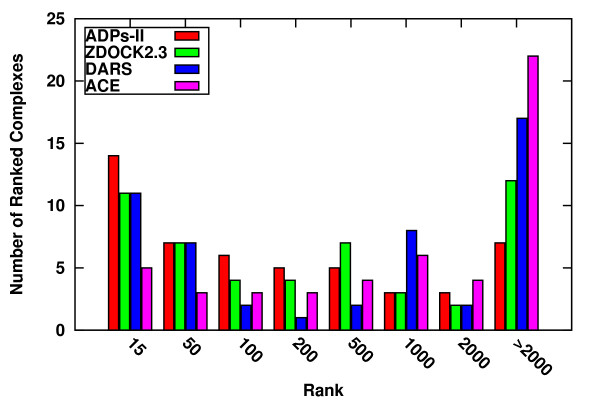
**Ranking success of ZDOCK2.3 decoys**. Four potentials, ADPs-II (red), ZDOCK2.3 (green), DARS (blue), and ACE (pink) were tested on of fifty complexes taken from ZDOCK2.3 decoys set. The ADPs-II potentials were subjected to a jackknife test (see Methods). The histogram shows the ranking distribution of the hits (best rank of near native decoy).

Next, we subjected the potentials to a jackknife test using the ZDOCK2.3 Decoys (6 degrees sampling) [5]. This test set contains 54000 predictions for each complex in the unbound state generated by the ZDOCK docking program. Each time ten complexes were selected randomly from dataset 1 (test complexes). Complexes homologous to the test complexes were removed from the training dataset and new sets of ADPs were developed (see Methods). The new potentials were used to rank order the decoys of the test set. This procedure was repeated five times to incorporate a total of fifty proteins. The average correlation coefficient between ADPs-II and the jackknifed potentials is 0.91 indicating the stability of the trained parameters. The ADPs-II performance was compared with two other potentials, the common Atomic Contact Energies (ACE) [13] and the newer DARS [15], as well with the Zdock2.3 scoring function. A hit was defined if the IRMSD ≤ 4Å, where IRMSD values were taken from the ZDOCK2.3 Decoys' supplied tables. The results have been summarized in Table 4, and depicted graphically in Figure 5.

**Table 4:** Ranking ZDOCK2.3 decoys (IRMSD ≤4Å) by the jackknife test

**Complex^a^**	**Rank^b^**			
	
	**ADPs-II^c^**	**Zdock2.3**	**DARS**	**ACE**
1AK4	-	-	950	1744
1B6C	95	16	9	178
1BVK	12	268	610	-
1DQJ	-	1490	-	-
1EWY	164	18	42	364
1FC2	484	-	24	797
1GHQ	-	-	-	1308
1HIA	21	653	122	-
1IJK	1131	-	368	-
1KLU	-	-	-	-
1AVX	5	46	6	59
1D6R	1840	567	-	-
1E96	39	399	42	490
1F34	13	26	666	5
1GCQ	1030	-	1057	1040
1GRN	210	102	-	-
1I4D	84	403	965	616
1KXP	91	7	1	200
1NSN	53	445	-	-
1SBB	-	-	-	-
1ACB	113	55	24	98
1AKJ	592	40	-	-
1AY7	120	100	-	-
1DFJ	37	1	-	-
1EAW	65	13	33	309
1FQ1	-	-	-	-
1GP2	4	389	58	1681
1I9R	184	14	589	-
1UDI	27	13	1	1
2PCC	203	193	821	120
1AHW	23	14	-	-
1BUH	14	-	555	-
1E6E	2	103	44	520
1ML0	10	1	9	32
1PPE	3	1	1	1
1QFW	544	74	-	-
1TMQ	9	126	12	22
1WEJ	3	42	1047	-
2JEL	307	86	76	579
7CEI	15	1	920	708
1ATN	-	-	-	-
1BJ1	6	18	18	85
1CGI	71	247	13	24
1F51	1	11	251	797
1KAC	121	1389	-	-
1KKL	283	-	1	222
1NCA	4	4	-	-
1QA9	687	-	-	-
2SNI	17	618	1	2
1RLB	48	302	5	7

a-Complex PDB code.

b-Rank of the lowest energy near-native decoy with IRMSD ≤4Å out of 54000 predictions.

c-Jackknife test (see Methods)

-- No near-native decoy identified among the 2000 best ranked decoys.

A near-native decoy was ranked in the top 100 places for twenty seven complexes using the ADPs-II, for twenty two using the Zdock2.3 scoring function, for twenty using DARS, and for eleven using ACE. The numbers of times the potentials failed to include a near-native case among the top 2000 raked decoys is: seven for the ADPs-II, twelve for the Zdock2.3 scoring function, seventeen for DARS, and twenty two for ACE. Thus, the ADPs-II outperform the other potentials on this set of decoys generated by a docking program different from the one used to generate the training decoys. The interfacial surface area of each complex was measured using the program Naccess [38]. No correlation was found between the size of the interfacial area and the success rate of the ADPs-II.

We also compared the capabilities of the ADPs-II and Zdock2.3 scoring functions to rank hits in the top ten places. In order to be consistent with the Zdock [5] studies, a hit is defined if the interfacial RMSD is ≤ 2.5Å. For thirty-nine out of the fifty complexes used in the Jackknife test such hits do exist in the Zdock2.3 decoy set. The results are presented in Table 5. A near-native decoy was ranked in the top 10 places for eight (20.5%) complexes using the ADPs-II, and for six (15.4%) using the Zdock2.3 scoring function.

**Table 5:** Ranking ZDOCK2.3 decoys (IRMSD ≤2.5Å) by the jackknife test

**Complex**	**Rank^a^**	
	
	**ADPs-II^b^**	**Zdock2.3**
1B6C	311	168
1BVK	-	-
1DQJ	-	-
1EWY	754	113
1IJK	1131	-
1AVX	955	449
1D6R	-	-
1E96	326	399
1F34	-	26
1GCQ	-	-
1GRN	492	807
1KXP	381	7
1NSN	-	445
1SBB	-	-
1AKJ	-	96
1AY7	236	-
1DFJ	720	1
1EAW	152	13
1I9R	329	90
1UDI	386	13
2PCC	-	-
1AHW	23	56
1BUH	16	-
1E6E	2	103
1ML0	10	1
1PPE	3	1
1QFW	1080	74
1TMQ	9	126
1WEJ	3	102
2JEL	307	86
7CEI	42	1
1BJ1	6	18
1CGI	-	-
1F51	1	11
1KAC	-	1523
1NCA	4	4
1QA9	-	-
2SNI	17	-
1RLB	48	302
**Top10**	**8 (20.5%)**	**6 (15.4%)**

a-Rank of the lowest energy near-native decoy with IRMSD ≤2.5Å out of 54000 predictions.

b-Jackknife test (see Methods)

--No near-native decoy identified among the 2000 best ranked decoys.

## Discussion and Conclusions

When trying to solve the set of inequalities derived from dataset 1 using the one step potentials (for either the ADPs or the SDPs) we encountered the state of infeasibility. That is, no set of parameters can satisfy all inequalities. These results indicate that neither the common functional form of atomic contact potentials [13,15] (18 atom types, see Table 1 and *r_cutoff _*values of 6.0 Å), nor the side chain-based potentials we designed [18], were adequate to discriminate at a high level between native and non-native docked complexes. We note that infeasibility was encountered for atomic contact potentials even when using a *r_cutoff _*value of 5.0 Å or 4.0 Å [18]. In the current study were able to overcome the problem by developing two-step potentials and doubling the number of parameters for the ADPs and the SDPs.

Two types of potentials were developed for protein-protein docking: one heavy atom based (ADPs-I), and one side-chain based (SDPs), using training datasets 1 and 2 for both cases. When we added dataset 3 to the training database we were still able to solve all derived constraints using the atomic model (ADPs-II) but not using the side-chain based model, in spite of the fact that the atomic level model has fewer parameters (342 types of interactions) than the side-chain based one (506 types of interactions). These results are in agreement with two other publications [17,23] showing that the discriminatory powers of atomic level potentials are superior to those of residue level potentials.

We have tested the tolerance of the resulting ADPs-II to structural changes between the bound and unbound structures under optimal conditions. That is when the complexes were included in the training set and the unbound decoys were generated by superimposing the unbound structures on the decoys generated in the bound state. This comparison enable us the test the effect of structural changes per se. The results (Figure 4) indicate that structural changes, measured by IRMSD of C^α^/all heavy atoms of more than 1.5Å/2.2Å, impair the ability of the potentials to rank a near native structure in the top 200 places.

Here we show that the ADPs-II outperforms two other contact potentials: the ACE and DARS. In addition, the ADPs-II outperforms the Zdock2.3 scoring function, which is a combination of desolvation energy (based on the ACE contact potential) electrostatics and shape complementarity [5]. The shape complementarity criterion alone was shown to have good ranking ability of near native structures [39]. Therefore, the performance of our ADPs-II in conjunction with shape complementarity and electrostatics is likely to improve.

The colour coded map of the potentials is shown in Figure 1a and 1b for ADPs-II and in Figure 1c for DARS. Analysis of the ADPs-II reveals that the first step potentials (r ≤ 4Å) are stronger in their magnitude than the second step potentials (4 < r ≤ 6Å). The average absolute value of the first and second step potentials is 1.25 and 0.4 respectively. The standard deviation of the ADPs-II is 1.88 and 0.79 for the first and second steps respectively and 0.74 for the DARS potentials. Thus, separating the potentials into two steps allows us to generate more varied potentials in the first step than in the second step that contains more information. While, the variability of the single step DARS potentials appear to be closer to that of the ADPs-II second step. The average interaction per atom type for the first and second step is displayed in Figure 2. Overall, the average interactions of the first step potentials are more repulsive than the second step potentials with hydrogen bond interactions being the most attractive ones in the first step potentials. On the other hand, in the second step potentials hydrophobic interactions are the most attractive ones. Comparing the ADPs-II with the DARS reveals some interesting differences. For example, CSγ-CSγ interaction is the most repulsive ADPs-II potential in both ranges whiles the most attractive DARS potential. We emphasize that our potentials were derived exclusively from transient complexes where the formation of Cys-Cys interactions are undesired, since these complexes are formed and detach from each other in response to specific biological conditions. The DARS potentials assign repulsive to neutral energies to all backbone interactions (atoms: N, Cα, C, and O), except for the Cα-Cα interaction, which is attractive. Our ADPs-II assign a relatively attractive energy to the C-N interactions at the close range and, therefore, captures more accurately H-bond interactions. Interestingly, and unlike the Cα-Cα interactions, GCα-GCα interactions also are assigned a large attractive energy. This may reflect the flexibly and compactness of the Gly side chains, allowing those to be in closer proximity with each other relative to other side chains.

Most computational approaches for protein-protein docking involve a tradeoff between accuracy and computational power. Therefore, a common scheme for protein docking is an initial on grid exhaustive search [5,8,40] using relatively simple scoring functions followed by off grid refinement and reranking stage using more elaborate energy functions. Several types of docking potentials were developed for these purposes. On one hand are the relatively simple one step potentials such as DARS and ACE, whereas on the other hand are the multiple step potential such as the ITScore-PP (step sizes of 0.2Å and R_cutoff _of 10Å) [23]. The latter potentials were shown to successfully rank the Zdock2.3 decoy (Huang and Zou[23], Table six), these potentials rank a near-native (IRMSD ≤ 2.5Å) in the top 10 places in 36.3% of the cases compared to a success rate of 20.5% for our ADPs-II (Table 5). The ITScore-PP scoring function is very appropriate for the reranking stage of the docked structure as it requires pre-minimization of the structures to remove clashes and its resolution (0.2Å) is much finer than the 1Å spacing usually used for the search grid. Here we developed an intermediate level of complexity potentials, closer in their functional form to the DARS and ACE than to the ITScore-PP. These potentials are likely to improve the initial on grid search step.

The main challenges in designing transient protein-protein docking potentials using the Boltzmann statistics are: (i) Compiling a none-redundant and representative dataset of protein complexes and, (ii) Setting a reference state. To develop the ACE, Zhang *et al*. used a set of protein folds as a learning dataset and their shuffled structures as a reference state [13]. Chuang *et al*. (DARS) used a set of transient and none-transient protein interfaces as a learning set and a large set of misdocked decoys as the reference state [15]. Using the LP approach we were able to train our potentials using a dataset of transient complexes *only *with no need to define a reference state. The ability to learn solely from transient complexes may add specificity toward this type of complexes. The above results show that the LP approach is a good alternative to the Boltzmann statistics in designing protein docking potentials.

## List of abbreviations

(ADPs): atom-based docking potentials; (DP): docking potential; (FP): false positive; (LP): linear programming; (RMSD): root mean square deviation; (IRMSD): interfacial RMSD; (SDPs): side-chain based docking potentials;   (TP): true positive.

## Authors' contributions

DT preformed the work and wrote the manuscript.

## Supplementary Material

Additional file 1 **Supplementary material**. Supplementary tables S1, S2 and S3 Click here for file
