# Supplementary material for: Designing coarse grained-and atom based-potentials for protein-protein docking
Source: BMC Struct Biol. 2010 Nov 15;10:40. doi: 10.1186/1472-6807-10-40 (PMC2996388; doi:10.1186/1472-6807-10-40)
Supplement: Additional file 1 — Supplementary material. Supplementary tables S1, S2 and S3 [file 1472-6807-10-40-S1.PDF]

Table S1a: Side chain-based Docking Potentials (SDPs) - step 1

	ALA	ARG	ASN	ASP	CYS	GLN	GLU	GLY	HIS	ILE	LEU	LYS	MET	PHE	PRO	SER	THR	TRP	TYR	VAL	NH	OC
ALA	-0.59	0.76	-0.02	-0.76	-0.53	-0.71	0.95	-0.64	-0.62	-0.90	-0.20	1.34	-0.75	-1.35	-0.54	0.02	-0.06	-1.42	-0.88	-1.61	0.09	0.27
ARG	0.76	0.07	0.26	-1.59	-0.35	0.21	-2.01	0.07	0.17	1.03	-0.60	1.40	-0.52	-1.01	-1.19	0.37	-0.48	-2.47	-0.96	-0.41	1.37	-1.33
ASN	-0.02	0.26	-0.34	-1.24	-0.37	-0.25	-0.83	0.24	-0.45	-0.48	0.02	-0.61	-0.58	0.93	0.27	-0.32	-0.58	-1.27	-1.60	0.26	0.19	-0.42
ASP	-0.76	-1.59	-1.24	0.96	-0.47	-0.43	0.18	0.12	-0.91	1.44	0.39	-2.15	-0.85	0.71	-0.07	-1.16	-0.70	-1.10	-0.37	-1.12	-0.15	0.38
CYS	-0.53	-0.35	-0.37	-0.47	10.00	1.55	1.38	0.28	-2.26	-0.20	-0.27	-1.03	-0.36	-0.35	1.81	0.18	3.04	3.27	1.14	-0.51	-0.63	1.40
GLN	-0.71	0.21	-0.25	-0.43	1.55	0.04	0.08	-0.65	-0.15	-1.61	-0.21	-0.64	-1.02	0.67	-0.30	-0.47	-0.41	-1.54	-1.11	-0.78	0.51	-0.44
GLU	0.95	-2.01	-0.83	0.18	1.38	0.08	1.69	-0.62	-0.90	-0.29	0.46	-1.69	-1.65	-0.95	-0.56	-0.93	-0.10	-1.09	-0.75	-0.42	0.07	-0.04
GLY	-0.64	0.07	0.24	0.12	0.28	-0.65	-0.62	-0.19	-1.26	-0.19	-0.59	-0.25	-0.69	0.75	0.75	0.40	0.13	-0.35	-0.70	-1.44	-0.76	0.60
HIS	-0.62	0.17	-0.45	-0.91	-2.26	-0.15	-0.90	-1.26	2.41	-1.84	-1.85	-0.08	-1.77	-2.61	-0.62	-0.33	-1.56	0.38	-1.77	-0.39	-0.01	-0.05
ILE	-0.90	1.03	-0.48	1.44	-0.20	-1.61	-0.29	-0.19	-1.84	-0.93	-1.54	-0.21	-0.89	-2.24	0.43	-0.64	-0.50	-2.84	-1.19	-0.85	1.36	-0.81
LEU	-0.20	-0.60	0.02	0.39	-0.27	-0.21	0.46	-0.59	-1.85	-1.54	-1.92	-1.20	-1.60	-1.92	-1.33	-0.60	-0.68	-0.78	-1.33	-1.52	0.16	-0.67
LYS	1.34	1.40	-0.61	-2.15	-1.03	-0.64	-1.69	-0.25	-0.08	-0.21	-1.20	0.40	-0.06	-0.34	-0.12	-0.67	0.61	-1.67	-1.22	-0.91	0.86	-0.71
MET	-0.75	-0.52	-0.58	-0.85	-0.36	-1.02	-1.65	-0.69	-1.77	-0.89	-1.60	-0.06	10.00	-2.98	-2.10	-1.30	-0.38	-0.04	-2.35	-2.25	0.68	-0.68
PHE	-1.35	-1.01	0.93	0.71	-0.35	0.67	-0.95	0.75	-2.61	-2.24	-1.92	-0.34	-2.98	-2.24	-1.70	0.57	-1.50	0.36	-1.66	0.16	0.03	-0.21
PRO	-0.54	-1.19	0.27	-0.07	1.81	-0.30	-0.56	0.75	-0.62	0.43	-1.33	-0.12	-2.10	-1.70	-2.64	-0.95	-0.16	-1.63	-1.40	-1.23	0.54	-0.15
SER	0.02	0.37	-0.32	-1.16	0.18	-0.47	-0.93	0.40	-0.33	-0.64	-0.60	-0.67	-1.30	0.57	-0.95	0.27	-0.23	-0.89	-0.88	0.42	0.02	-0.21
THR	-0.06	-0.48	-0.58	-0.70	3.04	-0.41	-0.10	0.13	-1.56	-0.50	-0.68	0.61	-0.38	-1.50	-0.16	-0.23	0.60	0.38	-0.14	-0.04	0.07	-0.03
TRP	-1.42	-2.47	-1.27	-1.10	3.27	-1.54	-1.09	-0.35	0.38	-2.84	-0.78	-1.67	-0.04	0.36	-1.63	-0.89	0.38	1.40	-2.23	0.32	-0.44	-0.46
TYR	-0.88	-0.96	-1.60	-0.37	1.14	-1.11	-0.75	-0.70	-1.77	-1.19	-1.33	-1.22	-2.35	-1.66	-1.40	-0.88	-0.14	-2.23	-1.63	-0.63	0.29	-0.20
VAL	-1.61	-0.41	0.26	-1.12	-0.51	-0.78	-0.42	-1.44	-0.39	-0.85	-1.52	-0.91	-2.25	0.16	-1.23	0.42	-0.04	0.32	-0.63	-1.45	0.31	-0.33
NH	0.09	1.37	0.19	-0.15	-0.63	0.51	0.07	-0.76	-0.01	1.36	0.16	0.86	0.68	0.03	0.54	0.02	0.07	-0.44	0.29	0.31	0.63	-0.02
OC	0.27	-1.33	-0.42	0.38	1.40	-0.44	-0.04	0.60	-0.05	-0.81	-0.67	-0.71	-0.68	-0.21	-0.15	-0.21	-0.03	-0.46	-0.20	-0.33	-0.02	-0.09

1. Step 1 is defined as: $r_1 \leq 4.5$ Å for backbone-backbone interactions, $r_1 \leq 5.5$ Å for backbone-side chain interactions, and $r_1 \leq 6.5$ Å for side chain-side chain interactions.

2. N(H) and O(C)2 represents the amide nitrogen (N), and the carbonyl oxygen (O) on the backbone.

Table S1b: Side chain-based Docking Potentials (SDPs) - step 2

	ALA	ARG	ASN	ASP	CYS	GLN	GLU	GLY	HIS	ILE	LEU	LYS	MET	PHE	PRO	SER	THR	TRP	TYR	VAL	NH	OC
ALA	-0.58	-0.40	0.02	0.85	0.54	0.04	-0.05	1.56	0.41	-0.24	0.12	0.94	-0.07	-0.15	1.75	0.16	-0.36	0.56	0.42	-0.17	-0.25	0.09
ARG	-0.40	0.72	0.37	-0.56	0.09	0.48	-0.64	-0.31	1.42	0.16	0.38	1.50	-0.42	1.11	0.70	0.43	0.36	-1.14	-0.20	-0.97	-0.07	-0.19
ASN	0.02	0.37	1.42	-0.06	1.13	0.20	-0.43	-0.04	0.81	0.47	0.38	-0.29	2.71	-0.64	-0.18	0.33	0.04	0.25	0.24	0.84	-0.20	0.24
ASP	0.85	-0.56	-0.06	0.22	-0.25	-0.14	-0.18	-0.09	-0.50	0.23	0.81	-0.24	0.20	-1.10	1.26	-0.27	-0.21	0.57	-0.42	-0.67	0.39	0.30
CYS	0.54	0.09	1.13	-0.25	1.10	-0.66	0.59	-0.13	6.43	0.12	0.86	0.33	0.55	-0.20	1.54	0.84	1.76	2.67	1.37	0.96	-1.20	-0.21
GLN	0.04	0.48	0.20	-0.14	-0.66	1.00	0.09	-0.11	0.85	0.42	-0.17	0.21	0.34	0.05	0.73	-0.45	0.27	-0.47	0.60	0.08	0.00	0.00
GLU	-0.05	-0.64	-0.43	-0.18	0.59	0.09	0.41	0.22	-0.12	1.18	0.33	-1.10	-0.26	0.65	1.02	-0.04	-0.51	0.85	0.45	1.43	0.37	-0.03
GLY	1.56	-0.31	-0.04	-0.09	-0.13	-0.11	0.22	-0.17	0.51	0.28	0.96	0.71	-0.74	0.45	0.06	0.53	0.42	-0.15	-0.32	0.67	-0.39	0.15
HIS	0.41	1.42	0.81	-0.50	6.43	0.85	-0.12	0.51	-0.19	-0.75	-1.22	0.78	4.03	0.98	0.07	0.39	1.14	1.54	-0.06	-0.51	0.00	0.24
ILE	-0.24	0.16	0.47	0.23	0.12	0.42	1.18	0.28	-0.75	-1.08	-1.28	0.26	-1.22	-0.78	1.53	0.82	0.54	0.30	-0.96	1.55	0.25	-0.56
LEU	0.12	0.38	0.38	0.81	0.86	-0.17	0.33	0.96	-1.22	-1.28	0.26	0.62	-0.12	0.37	-0.91	0.25	0.00	-0.01	0.08	-0.54	0.10	0.15
LYS	0.94	1.50	-0.29	-0.24	0.33	0.21	-1.10	0.71	0.78	0.26	0.62	1.74	0.78	0.22	-0.40	0.00	-0.13	-0.15	0.41	0.93	0.19	0.11
MET	-0.07	-0.42	2.71	0.20	0.55	0.34	-0.26	-0.74	4.03	-1.22	-0.12	0.78	10.00	0.74	0.81	0.12	0.00	0.30	1.16	6.00	0.05	0.43
PHE	-0.15	1.11	-0.64	-1.10	-0.20	0.05	0.65	0.45	0.98	-0.78	0.37	0.22	0.74	1.23	0.83	-0.13	0.35	-0.17	-0.66	-0.57	-0.38	-0.18
PRO	1.75	0.70	-0.18	1.26	1.54	0.73	1.02	0.06	0.07	1.53	-0.91	-0.40	0.81	0.83	-0.56	-0.31	0.06	1.90	-0.18	0.52	-0.20	0.74
SER	0.16	0.43	0.33	-0.27	0.84	-0.45	-0.04	0.53	0.39	0.82	0.25	0.00	0.12	-0.13	-0.31	-0.76	-0.46	-0.62	0.02	0.37	-0.09	0.23
THR	-0.36	0.36	0.04	-0.21	1.76	0.27	-0.51	0.42	1.14	0.54	0.00	-0.13	0.00	0.35	0.06	-0.46	0.01	-0.71	-0.38	-0.26	-0.36	0.28
TRP	0.56	-1.14	0.25	0.57	2.67	-0.47	0.85	-0.15	1.54	0.30	-0.01	-0.15	0.30	-0.17	1.90	-0.62	-0.71	-1.69	-0.59	0.16	0.14	-0.29
TYR	0.42	-0.20	0.24	-0.42	1.37	0.60	0.45	-0.32	-0.06	-0.96	0.08	0.41	1.16	-0.66	-0.18	0.02	-0.38	-0.59	2.07	-0.41	-0.31	-0.08
VAL	-0.17	-0.97	0.84	-0.67	0.96	0.08	1.43	0.67	-0.51	1.55	-0.54	0.93	6.00	-0.57	0.52	0.37	-0.26	0.16	-0.41	1.30	-0.04	0.45
NH	-0.25	-0.07	-0.20	0.39	-1.20	0.00	0.37	-0.39	0.00	0.25	0.10	0.19	0.05	-0.38	-0.20	-0.09	-0.36	0.14	-0.31	-0.04	-0.18	0.09
OC	0.09	-0.19	0.24	0.30	-0.21	0.00	-0.03	0.15	0.24	-0.56	0.15	0.11	0.43	-0.18	0.74	0.23	0.28	-0.29	-0.08	0.45	0.09	-0.24

1. Step 2 is defined as: $4.5 < r_2 \leq 6$ Å for backbone-backbone interactions, $5.5 < r_2 \leq 7$ Å for backbone- side chain interactions, and $6.5 < r_2 \leq 8.0$ Å for side chain-side chain interactions.

2. N(H) and O(C)2 represents the amide nitrogen (N), and the carbonyl oxygen (O) on the backbone.

Table S2a: Atomic Docking Potentials I (ADPs-I) step 1

	N	Cα	C	O	GCα	Cβ	KNζ	KCδ	DOδ	RNη	NNδ	RNε	SOγ	HNε	YCζ	FCζ	LCδ	CSγ
**N**	1.72	3.49	-4.60	0.26	1.05	-1.06	0.00	-1.06	-1.46	0.87	2.46	3.89	0.86	0.22	1.93	0.00	1.21	-1.15
**Cα**	3.49	4.96	0.86	-2.58	5.42	0.61	2.05	1.96	1.02	3.61	-0.53	3.77	1.30	0.99	-0.85	1.72	0.92	4.18
**C**	-4.60	0.86	6.27	1.16	3.92	2.30	2.44	2.21	1.40	-4.25	1.98	-1.82	1.04	1.88	2.06	1.29	0.70	7.05
**O**	0.26	-2.58	1.16	3.42	-4.88	-0.17	-2.39	-1.94	0.82	1.63	-2.09	-1.73	-1.46	-0.40	-0.99	0.66	-0.59	6.48
**GCα**	1.05	5.42	3.92	-4.88	0.71	2.47	4.83	-0.69	-0.08	5.26	-1.88	4.36	1.05	2.79	-3.50	0.38	1.44	-0.02
**Cβ**	-1.06	0.61	2.30	-0.17	2.47	2.23	-0.46	1.44	1.20	0.59	1.02	0.97	1.78	0.89	-0.12	-0.31	1.59	-0.26
**KNζ**	0.00	2.05	2.44	-2.39	4.83	-0.46	-3.14	10.00	-1.10	-0.52	2.45	2.36	0.53	0.74	-0.70	1.68	0.91	2.98
**KCδ**	-1.06	1.96	2.21	-1.94	-0.69	1.44	10.00	10.00	1.72	-0.71	-1.80	0.15	0.44	-1.31	1.42	-1.01	-0.60	1.55
**DOδ**	-1.46	1.02	1.40	0.82	-0.08	1.20	-1.10	1.72	0.38	-1.12	-0.17	0.79	-1.30	0.46	0.13	0.66	0.25	10.00
**RNη**	0.87	3.61	-4.25	1.63	5.26	0.59	-0.52	-0.71	-1.12	-0.70	-0.08	3.07	1.63	0.97	-1.42	1.88	0.27	-2.73
**NNδ**	2.46	-0.53	1.98	-2.09	-1.88	1.02	2.45	-1.80	-0.17	-0.08	0.21	2.80	0.85	0.95	2.92	0.54	1.06	2.82
**RNε**	3.89	3.77	-1.82	-1.73	4.36	0.97	2.36	0.15	0.79	3.07	2.80	0.94	-1.49	-0.62	1.64	-0.56	2.37	2.07
**SOγ**	0.86	1.30	1.04	-1.46	1.05	1.78	0.53	0.44	-1.30	1.63	0.85	-1.49	2.17	1.37	-0.08	0.86	0.86	5.69
**HNε**	0.22	0.99	1.88	-0.40	2.79	0.89	0.74	-1.31	0.46	0.97	0.95	-0.62	1.37	3.38	0.39	0.83	0.07	1.36
**YCζ**	1.93	-0.85	2.06	-0.99	-3.50	-0.12	-0.70	1.42	0.13	-1.42	2.92	1.64	-0.08	0.39	5.24	0.45	2.27	4.27
**FCζ**	0.00	1.72	1.29	0.66	0.38	-0.31	1.68	-1.01	0.66	1.88	0.54	-0.56	0.86	0.83	0.45	1.31	0.94	2.19
**LCδ**	1.21	0.92	0.70	-0.59	1.44	1.59	0.91	-0.60	0.25	0.27	1.06	2.37	0.86	0.07	2.27	0.94	2.88	0.44
**CSγ**	-1.15	4.18	7.05	6.48	-0.02	-0.26	2.98	1.55	10.00	-2.73	2.82	2.07	5.69	1.36	4.27	2.19	0.44	0.00

1. Step 1 is defined as $r_1 \leq 4$ Å

Table S2b: Atomic Docking Potentials I (ADPs-I) step 2

	N	Cα	C	O	GCα	Cβ	KNζ	KCδ	DOδ	RNη	NNδ	RNε	SOγ	HNε	YCζ	FCζ	LCδ	CSγ
**N**	0.29	-0.17	0.32	0.28	-0.23	-0.08	0.24	0.50	0.41	-0.85	0.85	0.60	0.13	0.73	1.46	-0.15	0.41	-2.72
**Cα**	-0.17	0.03	-0.97	-0.23	-1.46	-0.53	-0.32	-0.79	0.46	1.52	-0.58	0.56	0.35	-0.63	-2.06	-0.85	-1.10	1.06
**C**	0.32	-0.97	-0.64	-0.28	-0.29	0.30	0.04	-0.25	-0.87	-1.17	-0.80	0.30	-0.49	-0.42	0.53	0.26	-0.25	-3.73
**O**	0.28	-0.23	-0.28	0.87	0.50	0.40	0.38	1.29	-0.10	-0.20	-0.05	-1.54	-0.81	0.27	-0.32	-0.24	0.43	0.45
**GCα**	-0.23	-1.46	-0.29	0.50	-2.41	-0.28	-0.19	-0.71	0.82	1.10	0.06	1.15	2.35	-0.97	-1.63	-0.98	-1.99	-0.34
**Cβ**	-0.08	-0.53	0.30	0.40	-0.28	-0.93	0.32	1.16	0.46	-0.65	-0.12	0.10	0.19	0.03	-0.67	-0.10	-0.32	0.75
**KNζ**	0.24	-0.32	0.04	0.38	-0.19	0.32	-1.33	1.88	-0.66	-0.27	0.05	1.84	0.99	0.82	-1.30	-0.22	-0.88	1.37
**KCδ**	0.50	-0.79	-0.25	1.29	-0.71	1.16	1.88	-1.81	-0.97	-0.42	-1.00	1.66	-1.20	-0.49	1.32	-0.46	1.50	-2.08
**DOδ**	0.41	0.46	-0.87	-0.10	0.82	0.46	-0.66	-0.97	-0.17	-0.08	-0.38	-0.27	-0.06	-0.46	0.22	0.12	-0.03	-1.41
**RNη**	-0.85	1.52	-1.17	-0.20	1.10	-0.65	-0.27	-0.42	-0.08	0.43	-0.20	-0.05	-0.43	0.37	-0.45	0.12	-0.05	0.30
**NNδ**	0.85	-0.58	-0.80	-0.05	0.06	-0.12	0.05	-1.00	-0.38	-0.20	0.05	0.77	-0.20	-0.28	-0.65	-0.02	0.55	0.55
**RNε**	0.60	0.56	0.30	-1.54	1.15	0.10	1.84	1.66	-0.27	-0.05	0.77	-0.82	1.58	0.06	0.35	-1.06	0.87	0.37
**SOγ**	0.13	0.35	-0.49	-0.81	2.35	0.19	0.99	-1.20	-0.06	-0.43	-0.20	1.58	0.16	-0.48	0.12	-0.52	-0.07	-0.17
**HNε**	0.73	-0.63	-0.42	0.27	-0.97	0.03	0.82	-0.49	-0.46	0.37	-0.28	0.06	-0.48	1.18	-0.33	-0.48	-0.57	-1.62
**YCζ**	1.46	-2.06	0.53	-0.32	-1.63	-0.67	-1.30	1.32	0.22	-0.45	-0.65	0.35	0.12	-0.33	-1.26	-0.02	-0.43	2.69
**FCζ**	-0.15	-0.85	0.26	-0.24	-0.98	-0.10	-0.22	-0.46	0.12	0.12	-0.02	-1.06	-0.52	-0.48	-0.02	-0.38	-0.50	0.17
**LCδ**	0.41	-1.10	-0.25	0.43	-1.99	-0.32	-0.88	1.50	-0.03	-0.05	0.55	0.87	-0.07	-0.57	-0.43	-0.50	-1.08	1.95
**CSγ**	-2.72	1.06	-3.73	0.45	-0.34	0.75	1.37	-2.08	-1.41	0.30	0.55	0.37	-0.17	-1.62	2.69	0.17	1.95	10.00

1. Step 2 is defined as $4 < r_2 \leq 6$ Å

Table S3a: Atomic Docking Potentials II (ADPs-II) step 1

	**N**	**Cα**	**C**	**O**	**GCα**	**Cβ**	**KNζ**	**KCδ**	**DOδ**	**RNη**	**NNδ**	**RNε**	**SOγ**	**HNε**	**YCζ**	**FCζ**	**LCδ**	**CSγ**
**N**	-1.02	1.31	-2.88	0.57	5.54	-0.20	0.16	1.66	-1.27	0.08	0.19	3.23	1.37	0.94	-0.08	-0.12	0.58	-2.59
**Cα**	1.31	2.97	1.67	-1.70	0.91	1.04	1.70	1.66	1.22	2.50	-0.54	0.55	0.60	-0.23	-0.68	0.56	2.10	2.73
**C**	-2.88	1.67	2.97	0.41	3.28	1.19	0.69	1.15	0.14	-0.44	1.53	1.04	0.22	0.60	3.51	0.86	0.26	3.90
**O**	0.57	-1.70	0.41	1.53	-4.17	0.19	-2.09	-1.04	1.30	-0.87	-1.03	-0.33	-0.34	-0.11	-0.52	0.57	-0.32	1.06
**GCα**	5.54	0.91	3.28	-4.17	-5.90	1.12	4.10	4.68	0.39	1.99	-0.52	-1.48	1.69	0.26	-2.30	-0.57	1.36	0.76
**Cβ**	-0.20	1.04	1.19	0.19	1.12	1.27	0.61	0.06	0.06	0.19	0.67	1.10	0.49	0.80	0.31	0.19	0.52	-0.43
**KNζ**	0.16	1.70	0.69	-2.09	4.10	0.61	2.63	0.67	-0.68	0.21	1.77	2.75	0.30	1.22	-0.01	1.45	1.26	5.73
**KCδ**	1.66	1.66	1.15	-1.04	4.68	0.06	0.67	-1.02	1.07	-0.71	-1.43	-0.08	0.96	-1.25	0.89	0.63	-1.38	9.84
**DOδ**	-1.27	1.22	0.14	1.30	0.39	0.06	-0.68	1.07	0.11	-0.51	0.08	0.59	-0.56	-0.09	0.31	0.35	0.49	10.00
**RNη**	0.08	2.50	-0.44	-0.87	1.99	0.19	0.21	-0.71	-0.51	-0.45	0.11	1.53	0.54	0.11	-0.12	0.29	0.50	-1.09
**NNδ**	0.19	-0.54	1.53	-1.03	-0.52	0.67	1.77	-1.43	0.08	0.11	-0.12	2.15	0.49	0.02	0.84	0.51	-0.08	0.57
**RNε**	3.23	0.55	1.04	-0.33	-1.48	1.10	2.75	-0.08	0.59	1.53	2.15	-2.54	-0.05	0.32	0.33	-0.13	0.82	4.87
**SOγ**	1.37	0.60	0.22	-0.34	1.69	0.49	0.30	0.96	-0.56	0.54	0.49	-0.05	0.36	0.60	0.49	0.49	0.34	1.26
**HNε**	0.94	-0.23	0.60	-0.11	0.26	0.80	1.22	-1.25	-0.09	0.11	0.02	0.32	0.60	1.34	0.39	1.01	0.13	-0.59
**YCζ**	-0.08	-0.68	3.51	-0.52	-2.30	0.31	-0.01	0.89	0.31	-0.12	0.84	0.33	0.49	0.39	3.61	0.66	2.03	2.90
**FCζ**	-0.12	0.56	0.86	0.57	-0.57	0.19	1.45	0.63	0.35	0.29	0.51	-0.13	0.49	1.01	0.66	0.60	0.73	2.94
**LCδ**	0.58	2.10	0.26	-0.32	1.36	0.52	1.26	-1.38	0.49	0.50	-0.08	0.82	0.34	0.13	2.03	0.73	0.74	-2.03
**CSγ**	-2.59	2.73	3.90	1.06	0.76	-0.43	5.73	9.84	10.00	-1.09	0.57	4.87	1.26	-0.59	2.90	2.94	-2.03	10.00

1. Step 1 is defined as $r_1 \leq 4$ Å

Table S3b: Atomic Docking Potentials II (ADPs-II) step 2

	N	Cα	C	O	GCα	Cβ	KNζ	KCδ	DOδ	RNη	NNδ	RNε	SOγ	HNε	YCζ	FCζ	LCδ	CSγ
**N**	0.10	-0.34	0.31	0.25	-0.60	0.00	0.07	-0.18	0.22	0.04	0.47	0.03	0.15	-0.48	0.45	0.26	-0.07	0.47
**Cα**	-0.34	0.04	-0.31	0.03	-0.60	-0.52	-0.19	0.20	0.51	0.33	-0.33	-0.24	0.07	0.19	-1.38	-0.34	-0.27	-0.97
**C**	0.31	-0.31	-0.16	-0.39	0.68	0.07	0.19	0.30	-0.48	-0.38	-0.28	0.08	-0.22	-0.28	0.39	-0.29	0.02	-0.64
**O**	0.25	0.03	-0.39	0.45	0.29	0.36	-0.13	-0.10	-0.09	-0.17	0.11	-0.30	-0.32	0.23	0.13	-0.01	0.00	1.66
**GCα**	-0.60	-0.60	0.68	0.29	-0.13	0.01	-0.39	0.84	0.45	0.42	-0.09	-0.27	0.29	0.53	-1.42	-0.37	-1.19	-2.88
**Cβ**	0.00	-0.52	0.07	0.36	0.01	-0.29	0.17	1.08	0.10	-0.19	-0.07	0.12	-0.26	-0.14	-0.16	-0.24	-0.16	-0.72
**KNζ**	0.07	-0.19	0.19	-0.13	-0.39	0.17	-0.91	1.23	-0.43	0.52	-0.23	0.72	0.66	0.56	-0.49	-0.05	-0.12	-0.13
**KCδ**	-0.18	0.20	0.30	-0.10	0.84	1.08	1.23	1.05	-0.23	-0.43	-0.55	0.46	-0.48	-0.57	-0.57	-0.35	-0.93	2.73
**DOδ**	0.22	0.51	-0.48	-0.09	0.45	0.10	-0.43	-0.23	-0.02	-0.05	-0.03	-0.30	0.01	-0.06	0.23	-0.09	-0.06	-0.24
**RNη**	0.04	0.33	-0.38	-0.17	0.42	-0.19	0.52	-0.43	-0.05	-0.01	-0.30	0.23	0.13	0.48	-0.07	-0.06	0.15	-0.07
**NNδ**	0.47	-0.33	-0.28	0.11	-0.09	-0.07	-0.23	-0.55	-0.03	-0.30	-0.05	0.16	-0.09	0.24	-0.21	-0.03	0.41	-0.04
**RNε**	0.03	-0.24	0.08	-0.30	-0.27	0.12	0.72	0.46	-0.30	0.23	0.16	-0.60	-0.53	-0.36	-0.17	-0.01	-0.48	1.10
**SOγ**	0.15	0.07	-0.22	-0.32	0.29	-0.26	0.66	-0.48	0.01	0.13	-0.09	-0.53	0.29	-0.28	-0.11	-0.10	0.01	-0.49
**HNε**	-0.48	0.19	-0.28	0.23	0.53	-0.14	0.56	-0.57	-0.06	0.48	0.24	-0.36	-0.28	0.25	-0.15	-0.24	-0.16	1.98
**YCζ**	0.45	-1.38	0.39	0.13	-1.42	-0.16	-0.49	-0.57	0.23	-0.07	-0.21	-0.17	-0.11	-0.15	-0.49	-0.15	-0.19	0.52
**FCζ**	0.26	-0.34	-0.29	-0.01	-0.37	-0.24	-0.05	-0.35	-0.09	-0.06	-0.03	-0.01	-0.10	-0.24	-0.15	-0.21	-0.28	-0.55
**LCδ**	-0.07	-0.27	0.02	0.00	-1.19	-0.16	-0.12	-0.93	-0.06	0.15	0.41	-0.48	0.01	-0.16	-0.19	-0.28	-0.65	1.17
**CSγ**	0.47	-0.97	-0.64	1.66	-2.88	-0.72	-0.13	2.73	-0.24	-0.07	-0.04	1.10	-0.49	1.98	0.52	-0.55	1.17	10.00

1. Step 2 is defined as $4 < r_2 \leq 6$ Å
